# Validity of the Chinese Version of the Person-Environment Apathy Rating (PEAR-C) for Persons With Dementia

**DOI:** 10.1093/geroni/igab046.696

**Published:** 2021-12-17

**Authors:** Ying-Ling Jao, Ying-Yu Chao, Yo-Jen Liao, Diane Berish, An-Yun Yeh, Shang-Ti Chen

**Affiliations:** 1 Pennsylvania State University, University Park, Pennsylvania, United States; 2 Rutgers University, Newark, New Jersey, United States; 3 Penn State University, University Park, Pennsylvania, United States; 4 Hunter College of CUNY, New York, New York, United States; 5 National Dong-Hwa University, Shoufeng, Hualien, Taiwan (Republic of China)

## Abstract

Apathy is a prevalent neurobehavioral symptom in dementia. Despite that environmental stimulation plays a key role in apathy, it is often overlooked in assessment. The Person-Environment Apathy Rating (PEAR) scale is currently the only validated apathy scale for persons with dementia that addresses environmental stimulation and is only available in English. This project translated the PEAR scale into Mandarin Chinese and evaluated its content validity. The PEAR scale includes two subscales: PEAR-Environment and PEAR-Apathy. Each subscale includes six items. The PEAR scale translation and validation were conducted through a four-step process. First, the PEAR scale was translated from English into Chinese by two bilingual PhD-prepared researchers. Second, the two Chinese versions of PEAR (PEAR-C) were back-translated into English by another two bilingual PhD-prepared researchers. Third, three content experts reviewed the two translated scales and reconciled a final PEAR-C scale. Finally, these three experts individually rated the PEAR-C and evaluated its content validity item-by-item in two aspects: 1) content equivalence: appropriateness to use this scale in Chinese cultural setting, and 2) semantic equivalence: the scale remains the same meaning after translation. The content validity index (CVI) was calculated to sum the ratings across experts. The CVI of content equivalence for all items was 1.0 for both subscales. The CVI of semantic equivalence was 0.98 for the PEAR-Environment and 0.97 for the PEAR-Apathy. The PEAR-C shows substantial content validity. Its reliability and construct validity need further evaluation. This scale is promising to assess apathy for individuals with dementia in the Chinese-speaking community.

